# Supplementary material for: Structural shifts in food basket composition of rural and urban Philippines: Implications for the food supply system
Source: PLoS One. 2022 Mar 31;17(3):e0264079. doi: 10.1371/journal.pone.0264079 (PMC8970376; doi:10.1371/journal.pone.0264079)
Supplement: S1 Table — (DOCX) [file pone.0264079.s001.docx]

**S1 Table. Sub-group commodity budget shares, rural households.**

| Main commodity groups | Sub-commodity groups | 2006 | 2009 | 2012 | 2015 | 2018 | All |
| --- | --- | --- | --- | --- | --- | --- | --- |
| Rice | Well-milled and ordinary rice | 0.873 | 0.817 | 0.901 | 0.888 | 0.911 | 0.890 |
|  | Premium-quality rice | 0.127 | 0.183 | 0.099 | 0.112 | 0.089 | 0.110 |
| Other cereals | Maize | 0.152 | 0.138 | 0.110 | 0.122 | 0.111 | 0.121 |
|  | Other cereals (flour, cereal preparation, bread, pasta, and other bakery products) | 0.848 | 0.862 | 0.890 | 0.878 | 0.889 | 0.879 |
| Meat | Pork (fresh) | 0.368 | 0.363 | 0.403 | 0.372 | 0.348 | 0.363 |
|  | Chicken (fresh) | 0.344 | 0.349 | 0.326 | 0.341 | 0.337 | 0.338 |
|  | Beef (fresh) | 0.079 | 0.070 | 0.078 | 0.077 | 0.076 | 0.076 |
|  | Other meats (fresh, frozen, and preserved) | 0.217 | 0.227 | 0.201 | 0.217 | 0.247 | 0.230 |
| Fish | Fresh fish | 0.723 | 0.740 | 0.703 | 0.700 | 0.700 | 0.708 |
|  | Seafood (shrimp, crab, squid, and shell) | 0.012 | 0.012 | 0.058 | 0.068 | 0.073 | 0.055 |
|  | Other fish (dried/smoked and preserved) | 0.274 | 0.256 | 0.242 | 0.236 | 0.230 | 0.241 |
| Dairy products | Eggs (fresh and processed) | 0.390 | 0.394 | 0.328 | 0.326 | 0.319 | 0.339 |
|  | Milk (raw, condensed, powdered, cheese and curd, and others) | 0.610 | 0.606 | 0.672 | 0.674 | 0.681 | 0.661 |
| Fruits and vegetables | Fruits (fresh and preserved) | 0.296 | 0.285 | 0.307 | 0.301 | 0.315 | 0.306 |
|  | Vegetables (leaves, roots, potatoes and tubers, cassava, and others) | 0.704 | 0.715 | 0.693 | 0.699 | 0.685 | 0.694 |
| Miscellaneous | Edible oils | 0.068 | 0.079 | 0.134 | 0.128 | 0.138 | 0.121 |
|  | Sugar, jam, and honey | 0.105 | 0.107 | 0.214 | 0.213 | 0.226 | 0.193 |
|  | Non-alcoholic beverages (e.g., mineral water, soft drinks, fruit and vegetable juices) | 0.092 | 0.119 | 0.177 | 0.200 | 0.231 | 0.188 |
|  | Coffee, cocoa, and tea | 0.125 | 0.150 | 0.254 | 0.298 | 0.326 | 0.266 |
|  | Food products not elsewhere classified | 0.611 | 0.546 | 0.221 | 0.162 | 0.094 | 0.239 |

Notes: Authors’ computation based on FIES data. NFA stands for National Food Authority.
