# Supplementary material for: Structural shifts in food basket composition of rural and urban Philippines: Implications for the food supply system
Source: PLoS One. 2022 Mar 31;17(3):e0264079. doi: 10.1371/journal.pone.0264079 (PMC8970376; doi:10.1371/journal.pone.0264079)
Supplement: S2 Table — (DOCX) [file pone.0264079.s002.docx]

**S2 Table. Sub-group commodity budget shares, urban households.**

| Main commodity groups | Sub-commodity groups | 2006 | 2009 | 2012 | 2015 | 2018 | All |
| --- | --- | --- | --- | --- | --- | --- | --- |
| Rice | Well-milled and ordinary rice | 0.940 | 0.865 | 0.946 | 0.922 | 0.941 | 0.929 |
|  | Premium-quality rice | 0.060 | 0.135 | 0.054 | 0.078 | 0.059 | 0.071 |
| Other cereals | Maize | 0.063 | 0.053 | 0.058 | 0.068 | 0.068 | 0.064 |
|  | Other cereals (flour, cereal preparation, bread, pasta, and other bakery products) | 0.937 | 0.947 | 0.942 | 0.932 | 0.932 | 0.936 |
| Meat | Pork (fresh) | 0.352 | 0.350 | 0.367 | 0.364 | 0.343 | 0.350 |
|  | Chicken (fresh) | 0.287 | 0.304 | 0.320 | 0.324 | 0.325 | 0.317 |
|  | Beef (fresh) | 0.076 | 0.064 | 0.068 | 0.067 | 0.066 | 0.068 |
|  | Other meats (fresh, frozen, and preserved) | 0.291 | 0.287 | 0.250 | 0.251 | 0.271 | 0.271 |
| Fish | Fresh fish | 0.789 | 0.798 | 0.733 | 0.738 | 0.724 | 0.745 |
|  | Seafood (shrimp, crab, squid, and shell) | 0.011 | 0.012 | 0.077 | 0.080 | 0.089 | 0.067 |
|  | Other fish (dried/smoked and preserved) | 0.207 | 0.198 | 0.193 | 0.185 | 0.189 | 0.193 |
| Dairy products | Eggs (fresh and processed) | 0.407 | 0.405 | 0.295 | 0.292 | 0.298 | 0.325 |
|  | Milk (raw, condensed, powdered, cheese and curd, and others) | 0.593 | 0.595 | 0.705 | 0.708 | 0.702 | 0.675 |
| Fruits and vegetables | Fruits (fresh and preserved) | 0.344 | 0.331 | 0.354 | 0.349 | 0.342 | 0.343 |
|  | Vegetables (leaves, roots, potatoes and tubers, cassava, and others) | 0.656 | 0.669 | 0.646 | 0.651 | 0.658 | 0.657 |
| Miscellaneous | Edible oils | 0.059 | 0.067 | 0.113 | 0.106 | 0.120 | 0.103 |
|  | Sugar, jam, and honey | 0.071 | 0.066 | 0.166 | 0.162 | 0.188 | 0.151 |
|  | Non-alcoholic beverages (e.g., mineral water, soft drinks, fruit and vegetable juices) | 0.146 | 0.176 | 0.260 | 0.268 | 0.299 | 0.255 |
|  | Coffee, cocoa, and tea | 0.112 | 0.132 | 0.272 | 0.335 | 0.344 | 0.277 |
|  | Food products not elsewhere classified | 0.613 | 0.560 | 0.190 | 0.132 | 0.070 | 0.225 |

Notes: Authors’ computation based on FIES data. NFA stands for National Food Authority.
