# Supplementary material for: Structural shifts in food basket composition of rural and urban Philippines: Implications for the food supply system
Source: PLoS One. 2022 Mar 31;17(3):e0264079. doi: 10.1371/journal.pone.0264079 (PMC8970376; doi:10.1371/journal.pone.0264079)
Supplement: Attachment — Submitted filename: PONE_Reviewer_comments_R3_v1.0.docx [file pone.0264079.s003.docx]

| No. | Comment | Response |
| --- | --- | --- |
| 1 | The abstract should be more compact, indicating the goals of the research or the research question as well. | Thank you! The goals of the research and research question are stated in the abstract now.  Empirical evidence on the responsiveness and sensitivities of food consumption to its drivers is vital for conducting economic studies. Despite recent attempts to provide such estimates, much empirical work remains to be done considering the prevailing shifts in consumption trends in the Philippines. |
| 2 | However, these are missing from the paper anyway, so in the introduction, the research goals/questions should be indicated. | Thank you. The **introduction** is revised following your suggestion. |
| 3 | The literature review is well improved. I recommend extending it with these sources from Central Europe (where, due to the transition period, similar tendencies in food consumption could be observed): [https://ince.md/uploads/files/1455801255_maketa-es-2-2012.pdf](https://gcc02.safelinks.protection.outlook.com/?url=https%3A%2F%2Fince.md%2Fuploads%2Ffiles%2F1455801255_maketa-es-2-2012.pdf&data=04%7C01%7C%7C3b6280c362074559608908d99eeead3a%7Ced5b36e701ee4ebc867ee03cfa0d4697%7C0%7C0%7C637715571754123138%7CUnknown%7CTWFpbGZsb3d8eyJWIjoiMC4wLjAwMDAiLCJQIjoiV2luMzIiLCJBTiI6Ik1haWwiLCJXVCI6Mn0%3D%7C2000&sdata=MCzYe8FmyHcEzx7wPKZ76NElWtL6Dp1KBvklfOIJF1k%3D&reserved=0) (Consumption characteristics of branded meat products in Hungary: the value chain approach); [https://www.researchgate.net/publication/338431810_Evaluating_consumer_behavior_for_consumption_of_milk_and_cheese_in_Gjilan_Region_Kosovo](https://gcc02.safelinks.protection.outlook.com/?url=https%3A%2F%2Fwww.researchgate.net%2Fpublication%2F338431810_Evaluating_consumer_behavior_for_consumption_of_milk_and_cheese_in_Gjilan_Region_Kosovo&data=04%7C01%7C%7C3b6280c362074559608908d99eeead3a%7Ced5b36e701ee4ebc867ee03cfa0d4697%7C0%7C0%7C637715571754133096%7CUnknown%7CTWFpbGZsb3d8eyJWIjoiMC4wLjAwMDAiLCJQIjoiV2luMzIiLCJBTiI6Ik1haWwiLCJXVCI6Mn0%3D%7C2000&sdata=mI7QATYq%2BOoURK8I2mnQYXyRBISqSs3t04N6jvSTNpo%3D&reserved=0) | Thank you for your positive comment about improving the literature review.  While Central Europe and other regions, including Latin America and the Caribbean as well as Sub-Saharan Africa, are important for the current paper, we would like to keep the literature review coverage focused on Asia in the interest of time and space.  Follow-up papers will find it useful to cite the recommended papers. |
| 4 | It is not clear why the the data chapter is before the methodology description. I think it would be more logical to regard the data chapter as a part of the methodology | Thanks for the comment. We don’t have any preference for this, but the rationale behind assigning a separate chapter for the data part is to avoid the methodology from being too long.  Following your comment, we have placed the **Data** section after the **Methods** section. |
| 5 | I recommend the publication of this research paper after these minor improvements. | Thank you. |
